# Supplementary material for: Tuning phenylalanine fluorination to assess aromatic contributions to protein function and stability in cells
Source: Nat Commun. 2023 Jan 4;14:59. doi: 10.1038/s41467-022-35761-w (PMC9813137; doi:10.1038/s41467-022-35761-w)

Supplementary Information

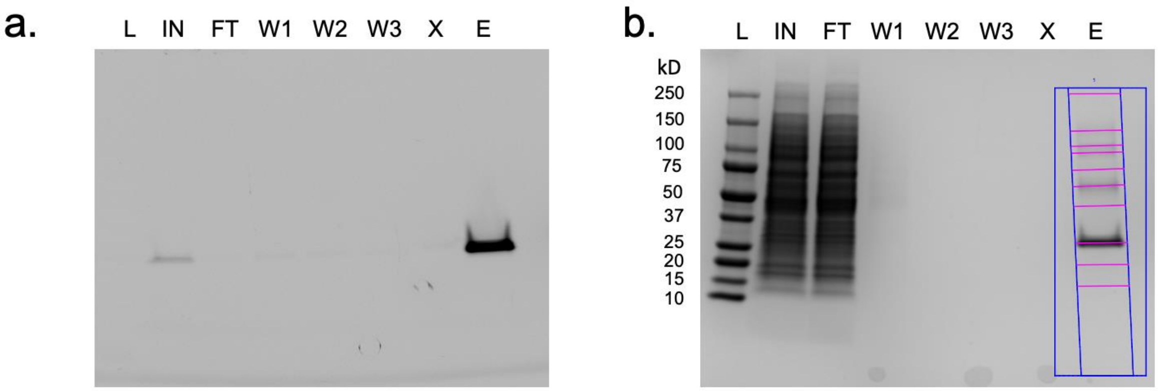

Supplementary Fig 1: Large scale prep and yield of sfGFP bearing penta-F phenylalanine at position N150 using $Phe_{X\text{-}D6}$. This large scale purification and gels were performed once. Purification was started from 3.26 g of HEK cells (31 10 cm dishes) **a.** SDS page gel imaged for GFP fluorescence before staining L= Ladder, IN= input, i.e., diluted lysate, FT= flowthrough, W1, W2, W3 = washes, X= empty, E = elution (diluted 1:1 in wash buffer). 30 μL of sample loaded per lane from 100 mL (IN/FT), 10 mL (Washes), 1.5 mL (E). Thus, ~1/50 of the elution was run in lane E. **b.** Coomassie stain of the same gel. Buffer exchange and concentration of elution resulted in 240 μL of 1.041 abs. Factoring the extinction coefficient of sfGFP_V5_HIS and a purity estimate (68%) from densitometry of the bands in Coomassie of E yields a total sfGFP estimate of 110.4 μg, or 34 μg/g of cells (wet pellet weight).

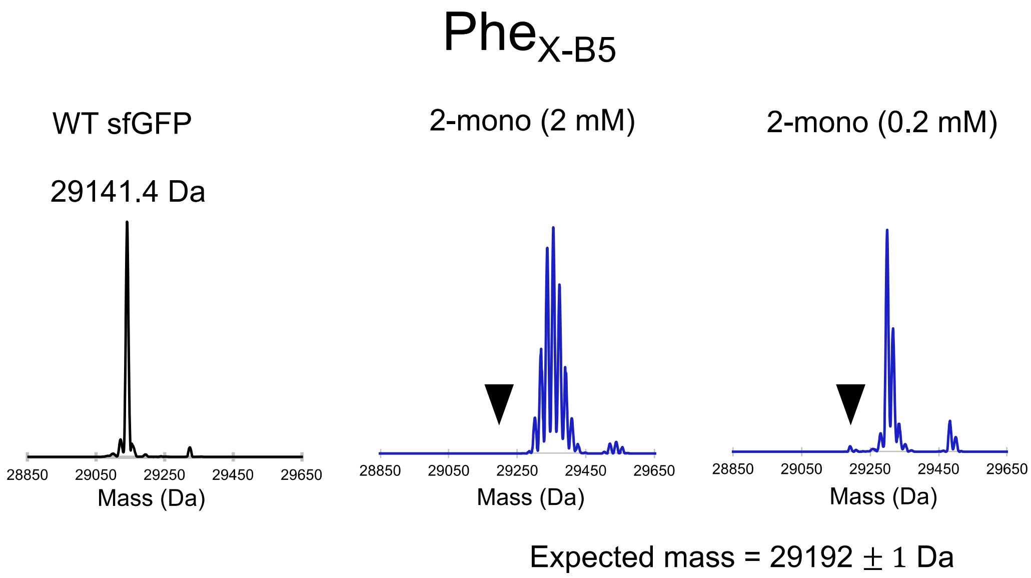

Supplementary Figure 2. Deconvoluted ESI mass spectra for sfGFP-N150TAG expressed with 2-mono fluoro phenylalanine and $Phe_{X\text{-}B5}$. Both 2 mM and 0.2 mM concentration of amino acid resulted in extensive incorporation of the amino acid at natural Phe codons, as indicated by masses at multiples of +18 Da. Signal at expected mass is absent in the 2 mM condition and a very small fraction (~1%) in 0.2 mM condition (black arrowheads).

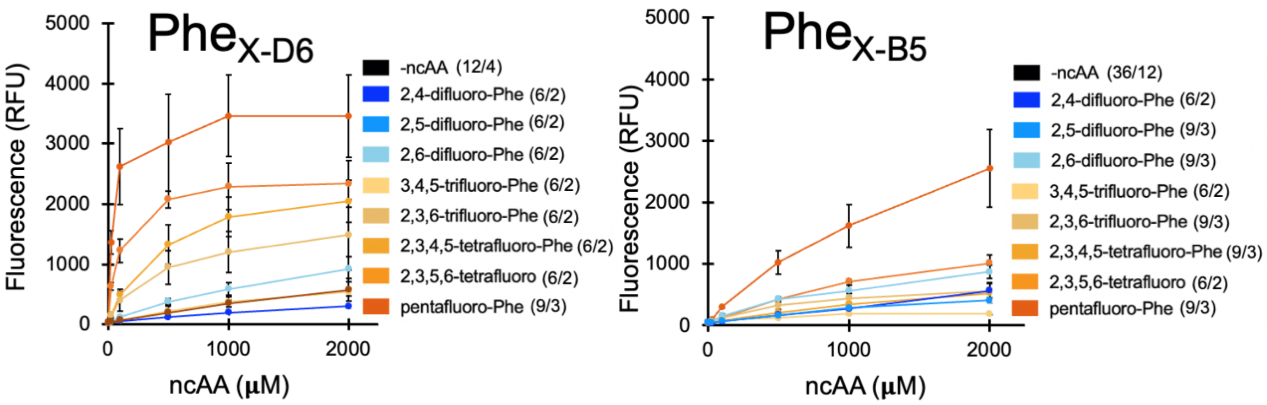

Supplementary Figure 3: GFP lysate values from HEKT cells co transfected with sfGFP150TAG and either Phe$_{X-D6}$ (left) or Phe$_{X-B5}$ (right) and harvested approximately 24 hours post transfection. In each case n values are given as X/Y where X equals the number of readings from Y independent experiments. Measure of center indicates mean ± standard deviation.

WT sfGFP

sfGFP N150_2,3,5,6F Phe (D6)

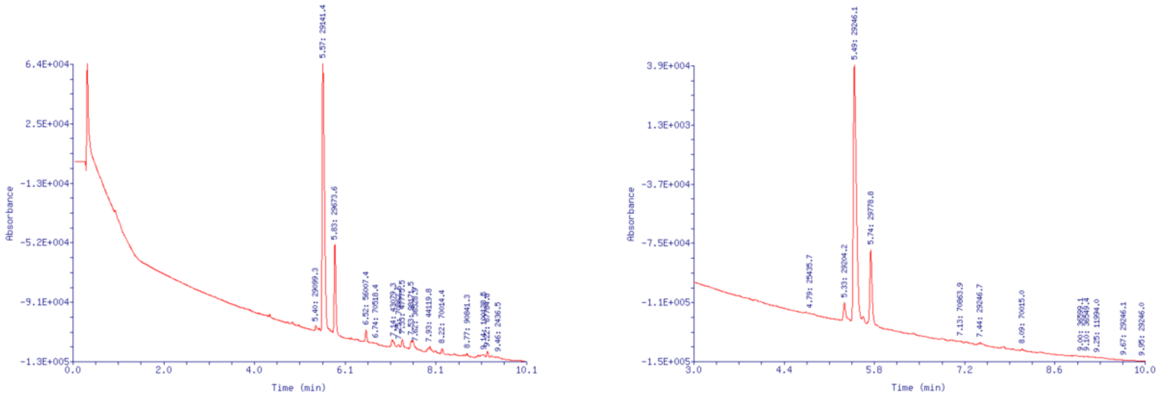

Supplementary Figure 4: UV absorbance of Liquid Chromatography of purified sfGFP samples. Both WT (left) and mutants (in this case Phe$_{X-D6}$ encoding 2,3,5,6 tetra-F Phe) eluted as 3 typical peaks for sfGFP, a dominant peak, as well as one at ~ -42 Da and a third at ~ + 532 Da. The deconvoluted mass spectra for the dominant (center) peaks are shown in the main figures.

	Activation					Steady State Inactivation				
	N	V_{mid}	SEM	d(x)	SEM	N	V_{mid}	SEM	d(x)	SEM
WT hNav1.5	5	-52.0	1.71	7.37	0.58	5	-97.8	0.85	6.76	0.45
F1486 (2,3,6F)	8	-52.8	1.56	7.31	0.66	8	**-104.5**	1.33	7.43	0.25

Supplementary Table 1: Gating parameters of $hNa_V1.5$ variants. **Bolded** = p value of 0.0014 compared to WT, two-sided t-test. P>0.24 for all other comparisons.

E. Coli expression- sfGFP_N150TAG_HIS

MVSKGEELFTGVVPILVELDGDVNGHKFSVRGEGEGDATNGKLTLKFICTTGKLPVPWPTLVTT
LTYGVQCFSRYPDHMKRHDFFKSAMPEGYVQERTISFKDDGTYKTRAEVKFEGDTLVNRIELK
GIDFKEDGNILGHKLEYNFNSH**(TAG)**VYITADKQKNGIKANFKIRHNVEDGSVQLADHYQQNTPI
GDGPVLLPDNHYLSTQSVLSKDPNEKRDHMVLLEFVTAAGITHGMDELYKGSHHHHHH

HEK Cell expression- sfGFP_N150TAG_V5_HIS

MVSKGEELFTGVVPILVELDGDVNGHKFSVRGEGEGDATNGKLTLKFICTTGKLPVPWPTLVTT
LTYGVQCFSRYPDHMKRHDFFKSAMPEGYVQERTISFKDDGTYKTRAEVKFEGDTLVNRIELK
GIDFKEDGNILGHKLEYNFNSH**(TAG)**VYITADKQKNGIKANFKIRHNVEDGSVQLADHYQQNTPI
GDGPVLLPDNHYLSTQSVLSKDPNEKRDHMVLLEFVTAAGITHGMDELYKGSGKPIPNPLLGLD
STHHHHHH

Supplementary Figure 5: Protein sequences of sfGFP variants used for expression and purification in this study.

Uncropped blots from data presented in Figure 7

Fig 7a hCFTR

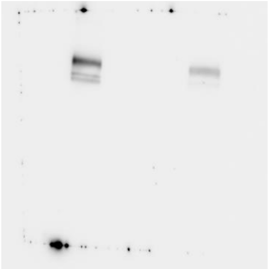

Fig 7b hNav1.5

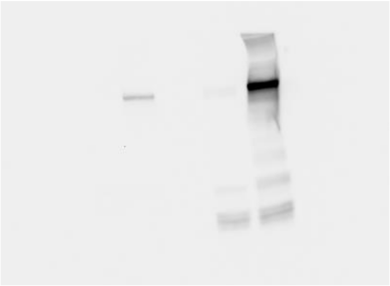

Fig 7a b-actin

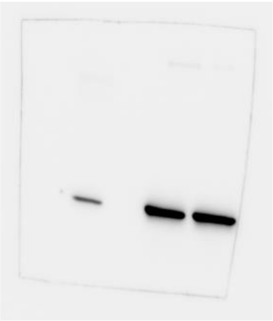

Fig 7b b-actin

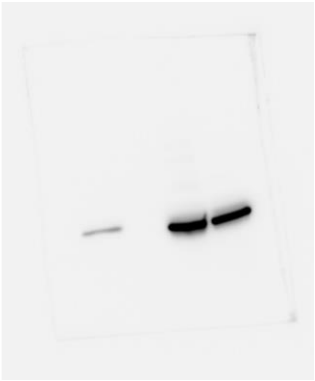

Supplement: Supplementary file 1 — Supplementary Information [file 41467_2022_35761_MOESM1_ESM.pdf]
