# Supplementary material for: Tuning phenylalanine fluorination to assess aromatic contributions to protein function and stability in cells
Source: Nat Commun. 2023 Jan 4;14:59. doi: 10.1038/s41467-022-35761-w (PMC9813137; doi:10.1038/s41467-022-35761-w)
Supplement: Supplementary file 3 — Reporting Summary [file 41467_2022_35761_MOESM3_ESM.pdf]

nature portfolio

Corresponding author(s): Christopher Ahern

Last updated by author(s): 12/13/2022

Reporting Summary

Nature Portfolio wishes to improve the reproducibility of the work that we publish. This form provides structure for consistency and transparency in reporting. For further information on Nature Portfolio policies, see our Editorial Policies and the Editorial Policy Checklist.

Statistics

For all statistical analyses, confirm that the following items are present in the figure legend, table legend, main text, or Methods section.

n/a	Confirmed	
☐	☒	The exact sample size (*n*) for each experimental group/condition, given as a discrete number and unit of measurement
☒	☐	A statement on whether measurements were taken from distinct samples or whether the same sample was measured repeatedly
☐	☒	The statistical test(s) used AND whether they are one- or two-sided<br>*Only common tests should be described solely by name; describe more complex techniques in the Methods section.*
☒	☐	A description of all covariates tested
☒	☐	A description of any assumptions or corrections, such as tests of normality and adjustment for multiple comparisons
☐	☒	A full description of the statistical parameters including central tendency (e.g. means) or other basic estimates (e.g. regression coefficient) AND variation (e.g. standard deviation) or associated estimates of uncertainty (e.g. confidence intervals)
☐	☒	For null hypothesis testing, the test statistic (e.g. *F*, *t*, *r*) with confidence intervals, effect sizes, degrees of freedom and *P* value noted<br>*Give P values as exact values whenever suitable.*
☒	☐	For Bayesian analysis, information on the choice of priors and Markov chain Monte Carlo settings
☒	☐	For hierarchical and complex designs, identification of the appropriate level for tests and full reporting of outcomes
☒	☐	Estimates of effect sizes (e.g. Cohen's *d*, Pearson's *r*), indicating how they were calculated

Our web collection on statistics for biologists contains articles on many of the points above.

Software and code

Policy information about availability of computer code

Data collection	Leica LASX 3, Biotek Gen5 3, Clampex 11, ImageLab 6
Data analysis	Fiji-ImageJ 2, Clampfit 10, ImageLab 6, Biotek Gen5 3, Origin Pro 11, Promass, Scaffold 5

For manuscripts utilizing custom algorithms or software that are central to the research but not yet described in published literature, software must be made available to editors and reviewers. We strongly encourage code deposition in a community repository (e.g. GitHub). See the Nature Portfolio guidelines for submitting code & software for further information.

Data

Policy information about availability of data

All manuscripts must include a data availability statement. This statement should provide the following information, where applicable:

- Accession codes, unique identifiers, or web links for publicly available datasets
- A description of any restrictions on data availability
- For clinical datasets or third party data, please ensure that the statement adheres to our policy

Source data are available within our submitted source data file.

Human research participants

Policy information about studies involving human research participants and Sex and Gender in Research.

Reporting on sex and gender	N/A
Population characteristics	N/A
Recruitment	N/A
Ethics oversight	N/A

Note that full information on the approval of the study protocol must also be provided in the manuscript.

Field-specific reporting

Please select the one below that is the best fit for your research. If you are not sure, read the appropriate sections before making your selection.

☒ Life sciences ☐ Behavioural & social sciences ☐ Ecological, evolutionary & environmental sciences

For a reference copy of the document with all sections, see nature.com/documents/nr-reporting-summary-flat.pdf

Life sciences study design

All studies must disclose on these points even when the disclosure is negative.

Sample size	No prior sample size calculation was performed. The sample sizes chosen are consistent with expectations in the field and see, for example, PMID #30733386 for electrophysiology and PMID #20082521 for characterization of Pyl-based synthetases.
Data exclusions	During analysis, three sodium channel whole cell recordings were excluded. Two were excluded because their exceptionally high channel density caused obviously apparent voltage clamp errors (clamp escape), and one was excluded because it had very high leak (>10% of peak current). The rest of the recordings analyzed were well clamped and had low leak (<5% of peak current).
Replication	Instances of replication in the study are described in the methods and figures. In addition, we note that the key result of competence of D6 and B5 synthetases to produce sfGFP with a fluoro phe analog specifically at N150 (as verified by mass spec) were done at least once each by scientists at OSU and UI independently. Due to prohibitively high cost, mass spec experiments were done once. However, these experiments were performed and analyzed by independent (off-site) facilities (Novatia, Inc. for intact sfGFP and UI proteomics core for Nav 1.5) and residual samples were saved for future reanalysis if necessary.
Randomization	In all cases this study utilized clonal (genetically identical) cell lines, handled and dispensed identically and in parallel for experiments
Blinding	Blinding was not conducted in this study; samples were labeled with actual identities for efficiency.

Reporting for specific materials, systems and methods

We require information from authors about some types of materials, experimental systems and methods used in many studies. Here, indicate whether each material, system or method listed is relevant to your study. If you are not sure if a list item applies to your research, read the appropriate section before selecting a response.

Materials & experimental systems

n/a	Involved in the study	
☐	☒	Antibodies
☐	☒	Eukaryotic cell lines
☒	☐	Palaeontology and archaeology
☒	☐	Animals and other organisms
☒	☐	Clinical data
☒	☐	Dual use research of concern

Methods

n/a	Involved in the study	
☒	☐	ChIP-seq
☒	☐	Flow cytometry
☒	☐	MRI-based neuroimaging

Antibodies

Antibodies used	AC-15/HRP (conjugated beta actin antibody-Novus Biologicals); ab596 (anti CFTR antibody- Cystic Fibrosis Foundation); D9J7S

Antibodies used	(anti Nav 1.5 antibody- Cell Signaling Inc). Catalogue # for AC-15 HRP from Novus is NB600-501H, the CFTR AB 596 is obtained by its name specifically, and cat # for the anti-nav D9J7S is #14421S.
Validation	All three antibodies above are validated on their websites and have been used in numerous studies. The respective websites are https://www.novusbio.com/products/beta-actin-antibody-ac-15_nb600-501, https://cftrantibodies.web.unc.edu/, and https://www.cellsignal.com/products/primary-antibodies/nav1-5-d9j7s-rabbit-mab/14421

Eukaryotic cell lines

Policy information about cell lines and Sex and Gender in Research

Cell line source(s)	ATCC CRL-3216
Authentication	Cell lines were not authenticated- they were obtained directly from ATCC and used.
Mycoplasma contamination	Cells were not tested for mycoplasma contamination
Commonly misidentified lines (See ICLAC register)	NO
